# pXOOY: A dual-function vector for expression of membrane proteins in *Saccharomyces cerevisiae* and *Xenopus laevis* oocytes

**DOI:** 10.1371/journal.pone.0281868

**Published:** 2023-02-21

**Authors:** Victoria Amstrup Vold, Sebastian Glanville, Dan Arne Klaerke, Per Amstrup Pedersen

**Affiliations:** 1 Department of Biology, University of Copenhagen, Copenhagen Ø, Denmark; 2 Department of Veterinary and Animal Sciences, University of Copenhagen, Frederiksberg, Denmark; Western University, CANADA

## Abstract

On the quest for solving structures of membrane proteins by X-ray crystallography or cryo-EM, large quantities of ultra-pure protein are a paramount prerequisite. Obtaining enough protein of such high standard is not a trivial task, especially for difficult-to-express membrane proteins. Producing membrane protein for structural studies is often performed in *Escherichia coli* or *Saccharomyces cerevisiae* and is frequently complemented with functional studies. Ion channels and electrogenic receptors are traditionally studied in terms of their electrophysiological behavior, which cannot be performed in neither *E*. *coli* nor yeast. Therefore, they are frequently characterized in mammalian cells or in *Xenopus laevis* oocytes. To avoid generating two different plasmids, we here describe the construction of a dual-function plasmid, pXOOY, for membrane protein production in yeast and for electrophysiology in oocytes. pXOOY was constructed such that all elements required for oocyte expression were copied from the dual *Xenopus*-mammalian vector pXOOM and meticulously introduced into the high-yield yeast expression vector pEMBLyex4. pXOOY is thus designed to preserve the high yield of protein from pEMBLyex4 while simultaneously permitting *in vitro* transcription for expression in oocytes. We evaluated the performance of pXOOY by comparing expression of two yeast codon optimized human potassium channels, ohERG and ohSlick (Slo2.1) from pXOOY to expression of these channels obtained from the reference vectors pEMBLyex4 and pXOOM. Our proof-of-concept study indicates that accumulation in PAP1500 yeast cells was higher when the channels were expressed from pXOOY, which was verified both qualitatively and quantitatively. Two-electrode voltage clamp measurements in oocytes showed that the pXOOY constructs encoding ohERG and ohSlick gave currents with full preservation of electrophysiological characteristics. Our results show that it is possible to design a dual-function *Xenopus*-yeast vector without compromising expression in yeast and simultaneously maintaining channel activity in oocytes.

## Introduction

Upon purifying membrane protein destined for structural studies it is essential to ensure that the protein is expressed and purified in its functional form. By doing so, we can more confidently say that the resolved structure is related to the functional protein. Furthermore, the protein structure usually predicts important amino acids whose function can be analyzed experimentally. However, functional characterization of a membrane protein is not necessarily performed in the same organism as used for protein production [1, 2]. Production of many membrane proteins is carried out in *E*. *coli* or *Saccharomyces cerevisiae* as these organisms give high yields of recombinant proteins, and their maintenance is associated with relatively low efforts and costs. Despite these advantages, membrane protein functionality assays are limited in these organisms. Ion channels and electrogenic receptors are often characterized by electrophysiological parameters such as gating mechanisms, ion selectivity as well as activation and inactivation kinetics [3]. The small diameters of *E*. *coli* and yeast cells as well as their cell walls make these organisms unsuitable for a number of methods used to characterize membrane protein including electrophysiological measurements. Characterization of ion channels and electrogenic receptors is therefore often performed in either mammalian cells or *Xenopus laevis* oocytes by means of patch clamp or two-electrode voltage clamp techniques. Consequently, these proteins are traditionally expressed from two different plasmids in order to both purify and functionally characterize them. Until this point, our labs have performed characterization studies on ion channels and transporters expressed from the plasmid pXOOM in *Xenopus laevis* oocytes [4] and simultaneously purified the same proteins expressed from the pEMBLyex4 plasmid in *S*. *cerevisiae* [5].

In this study, we have attempted to bridge the gap between the two expression systems by constructing a plasmid that permits ion channel expression in yeast as well as *Xenopus laevis* oocytes. Our study demonstrates that it is possible to construct a single dual-function plasmid that enables expression of ion channels in both *Xenopus laevis* oocytes and in *S*. *cerevisiae*. Our PAP1500 yeast expression system [6] will serve as a protein production and purification platform and expression in oocytes offers the opportunity to compare the electrophysiological characteristics of the wild-type protein as well as amino acid substituted versions.

As a proof-of-concept, we chose the two voltage-gated K^+^ channels, hERG and Slick expressed with a TEV-yEGFP-His_10_ tag from yeast codon optimized cDNA. The hERG channel is a voltage-gated delayed rectifying K^+^ channel highly expressed in heart tissue [7] where it is responsible for repolarization of the cardiomyocytes after an action potential [8]. The hERG channel is activated by a moderate membrane depolarization and inactivated by a positive membrane potential, which results in a very district I-V relationship. Slick (Slo2.1) belongs to the family of high-conductance K^+^ channels and it is expressed throughout the brain where it is thought to regulate neuronal excitability. Slick is activated by intracellular Na^+^ and exhibits low voltage-dependency resulting in an almost linear I-V relationship [9–12].

During the construction of pXOOY, we prioritized the DNA elements required for yeast expression and used yeast codon optimized ion-channel cDNAs in order to maintain the high expression levels from pEMBLyex4. Since the oocyte expression system is based on injection of polyA RNA generated by T7 RNA polymerase-mediated *in vitro* transcription, it was necessary to introduce a T7 promoter upstream of the open reading frame. To ensure poly-adenylation of the RNA transcript we exchanged the yeast 3’UTR with the *Xenopus laevis* beta-globin 3’UTR from pXOOM. This 3’UTR in pXOOM has been modified such that it incorporates a synthetic polyA tail into the RNA transcript, which is important to enhance its stability and translatability in the oocytes [13].

## Materials and methods

### Strains

Plasmid assembly by homologous recombination was carried out in *S*. *cerevisiae* strain PAP1500 (*MATα ura3-52 trp1*::*GAL10-GAL4 lys2-801 leu2Δ1 his3Δ200 pep4*::*HIS3prb1Δ1*.*6R can1 GAL*) [6]. Plasmid rescue from yeast total DNA was performed in Omnimax F′ [*proAB+ lacIq lacZΔM15 Tn10(TetR) Δ(ccdAB)] mcrA Δ(mrr-hsdRMS-mcrBC) φ80lacZΔM15 Δ(lacZYA-argF*) *U169 endA1 recA1 supE44 thi-1 gyrA96 (NalR) relA1 tonA panD*] (Thermo Fisher, USA). *Xenopus laevis* oocytes were purchased from EcoCyte Biosciences, Germany.

### Plasmid construction by homologous recombination in yeast

All cDNAs for cloning were purchased as yeast codon optimized DNA fragments from GenScript, USA. Codon optimized KCNH2 encoding hERG (UniProt ID Q12809-1) was purchased as one fragment whereas codon optimized KCNT2 encoding human Slick (UniProt ID Q6UVM3-1) was purchased as two fragments. The yEGFP DNA fragment was PCR amplified from a yeast codon optimized version [14]. The dual yeast and *Xenopus laevis* expression cassette was purchased as one DNA fragment from TAG Copenhagen, Denmark. The nucleotide sequence of the expression cassette can be found in S1 File. All PCR reactions were performed using Accupol DNA polymerase (Amplicon, Denmark) and the products were analyzed on 0.8% agarose gels. The PCR primers all contained 5’ extensions designed for homologous recombination to either pEMBLyex4 or to an adjacent PCR fragment [15]. PCR primers were furthermore designed to have a melting temperature, Tm, of 58°C using the formula Tm = 2 x (A+T) + 4 x (C+G) [16]. All primers were purchased from TAG Copenhagen, Denmark, and their nucleotide sequences can be found in S1 Table. The pXOOY plasmid is available from Addgene with deposit number 196449.

### Construction of ohERG and ohSlick expression plasmids by homologous recombination

pEMBLyex4 was linearized overnight at 37°C with *Bam*HI, *Hin*dIII, *Sal*I and *Xho*I (Thermo Fisher Scientific, USA) and pXOOY was linearized overnight at 37°C with *Bam*HI and *Hin*dIII (Thermo Fisher Scientific, USA) and both were subsequently heated to 80°C for 10 minutes for restriction enzyme inactivation. PAP1500 yeast cells were transformed with overlapping PCR fragments according to [17] and transformed cells selected on minimal medium containing 2% glucose as well as lysine (20mg/L) and leucine (30mg/L). For rescuing assembled plasmids, total yeast DNA was purified from 10mL cells lysed with lyticase enzyme using the NucleoSpin plasmid DNA purification kit (Macherey-Nagel, Germany).

### Expression in yeast

Small-scale production of ohERG-TEV-yEGFP-His_10_ and ohSlick-TEV-yEGFP-His_10_ in PAP1500 cells was performed as described in [18]. In short, yEGFP-positive PAP1500 transformants were inoculated in 5mL minimal medium containing lysine and leucine and grown overnight at 30°C. 200μl of this culture was transferred to 5mL minimal medium with lysine only and grown overnight. All 5mL were then transferred to 50mL minimal medium supplemented with lysine and grown overnight at 250rpm at 30°C. 1L YP expression medium (10g/L yeast extract, 20g/L peptone) containing 0.5% glucose and 7% glycerol was inoculated to an OD_450_ of 0.05 with the 50mL culture. When OD_450_ reached 1.5, the culture was transferred to 15°C and allowed to thermoequilibrate before protein production was induced with galactose dissolved in YP medium with 7% glycerol to a final concentration of 2%. Growth was followed by measuring OD_450_ and expression was monitored by measuring GFP fluorescence of 1 OD_450_ unit once a day for 5 days (at 0hrs, 24hrs, 48hrs, 72hrs, 96hrs and 120hrs after induction with galactose). GFP fluorescence was measured in a white 96-well microplate using a Fluoroskan Ascent fluorometer (Thermo Scientific, USA) with excitation at 485 nm and emission at 520 nm. At each time point, 50mL culture was harvested, and yeast membranes were obtained in a small-scale purification according to [6] with a few modifications. Briefly, cells from the 50mL samples were harvested and resuspended in 0.8mL ice cold lysis buffer (25mM Tris-HCl, 1mM EGTA, 1mM EDTA, 1M glycerol, 1M KCl, 1mM PMSF, 1μg/mL leupeptin (L), 1μg/mL pepstatin (P), 1μl/mL chymostatin (C) and 5mM β-mercaptoethanol at pH 7.5) and homogenized by glass-bead homogenization six times 1 minute. The cell homogenate was subsequently centrifuged at 3,000g for 10 minutes at 4°C and membranes pelleted from the resulting supernatant by ultracentrifugation at 200,000g for 30 minutes also at 4°C.

Protein concentrations in crude membrane samples were measured in a BCA assay according to the manufacturer’s recommendations [19]. Crude membrane volumes corresponding to 25μg protein were denatured for 15 minutes at RT in SDS sample buffer containing 1mM PMSF, 1μg/mL L, P, C and 5mM β-mercaptoethanol and analyzed in 10% SDS-PAGE gels. In-gel fluorescence from unstained gels was visualized using the Image Quant LAS-4000 system (GE Healthcare, USA) according to [20].

GFP fluorescence in 1 OD_450_ was averaged across the four replicates performed for each construct and plotted as the average ± S.D. using Prism 9 (GraphPad Software, USA). Prism 9 was also used to compare expression from the original pEMBLyex4 vector to the new pXOOY vector in a two-way ANOVA test.

### Transformation and plasmid purification from *E*. *coli*

Omnimax *E*. *coli* cells were transformed with total yeast DNA to rescue plasmids assembled by homologous recombination in yeast according to [21]. The cells were grown in SOC medium for 1hr after transformation for phenotypic expression and then transferred to LB agar plates containing 8μg/mL tetracycline and 100μg/mL ampicillin. Plasmid DNA was midi-prepped from 200mL cell culture using the NucleoBond Xtra Midi kit (Macherey-Nagel, Germany). All plasmids were fully sequenced and cryostocks were saved of each sequenced clone. Primers used for sequencing are found in S2 Table.

### Expression in *Xenopus laevis* oocytes

All pXOOY plasmids were linearized with *Nhe*I (New England Biolabs, USA) overnight at 37°C. Linearized plasmid DNA was purified using the High Pure PCR Purification kit (Roche, Switzerland). cRNA from all plasmids was generated by *in vitro* transcription using the mMessage mMachine T7 kit (ThermoFisher Scientific, USA) and purified using MEGAclear kit (Ambion, USA). Defolliculated *Xenopus laevis* oocytes stage V-VI were each injected with 50.6nl containing 25ng polyA RNA encoding either ohSlick-TEV-yEGFP-His_10_ or ohERG-TEV-yEGFP-His_10_. Injections were performed using a Nanoject microinjector (Drummond Scientific, USA). Oocytes were kept at 19°C in fresh Kulori medium (90 nM NaCl, 1 mM KCl, 1 mM MgCl_2_, 1 mM CaCl_2_, 5 mM HEPES, pH 7.4) for 5–6 days prior to electrophysiological measurements.

### Electrophysiology

Currents were measured using the oocyte voltage clamp amplifier OC-725B (Warner Instruments, USA) in a two-electrode voltage clamp setup. Sharp glass micropipettes were pulled from WPI TW120-3 glass capillaries with filament (World Precision Instruments, USA) on a vertical PC10 puller (Narishige, Japan) and backfilled with 3M KCl. Reference Ag/AgCl coated silver wires were placed in a separate 3M KCl reference bath, which was connected to the recording bath by 3M KCl agar bridges. Oocytes were kept in cold Kulori during all recordings. The voltage dependence of ohERG-TEV-yEGFP-His_10_ activity was investigated using a step protocol with a holding potential of -90mV and 2 s test potentials from -100mV to +40mV with 20mV increments. To ensure channel recovery from inactivation, a 1s step at -120mV was included after each test potential [22]. The voltage dependence of ohSlick-TEV-yEGFP-His_10_ activity was studied using a step protocol with 1 s test potentials ranging from -100mV to +80mV also with 20mV increments from a holding potential of -80mV [23].

Data acquisition was performed in Clampex (Molecular Devices, USA) and analysis performed in Clampfit (Molecular Devices, USA) as well as in Prism 9 (GraphPad Software, USA). I-V curves were constructed from 11 or 13 measurements for Slick and hERG currents, respectively, and represented as the average ± S.E.M.

### Bioimaging in yeast

Expression and localization of ohERG-TEV-yEGFP-His_10_ and ohSlick-TEV-yEGFP-His_10_ in yeast cells were visualized at 1,000x magnification by excitation at 488nm and emission at 520nm using a Nikon Eclipse E600 fluorescence microscope (Nikon Instruments Inc., USA) coupled to a Magnafire camera model S99802 (Optronics, USA).

### Bioimaging in oocytes

Localization of ohERG-TEV-yEGFP-His_10_ or ohSlick-TEV-yEGFP-His_10_ in oocytes was visualized at 10x magnification using a Leica TCS SPE DMI400B multipoint confocal-spinning disc microscope (Leica Microsystems, Germany). The dye FM 4–64 (ThermoFisher Scientific, USA) was included as a control dye for the plasma membrane [24]. Both yEGFP and FM 4–64 were excited at 488 nm using an EL6000 laser (Leica Microsystems, Germany). Fluorescence was detected using two different filters, 491–570 nm for yEGFP and 591–670 nm for FM 4–64. Prior to imaging, the oocytes were incubated for 1 hour in ice cold Kulori containing 2 μM FM 4–64 on ice to slow dye internalization. After incubation, the oocytes were removed from the FM 4–64 solution, washed in ice cold Kulori and subsequently transferred to ice cold Kulori in a homemade imaging chamber. The oocytes were oriented such that both animal and vegetal pole were visible. No noticeable difference or pattern in ion channel localization was observed in either construct. Leica LAS X software (Leica Microsystems, Germany) was used for image acquisition of the two different dyes. Fiji software was used for image processing to merge the channels using the two different filters [25].

### Bioinformatics

Information on codon frequencies in *S*. *cerevisiae* and *Xenopus laevis* were based on genomic DNA RefSeq assemblies and retrieved using the online Codon Codon-Pair Usage Table (CoCoPUT) database. The resulting graph was adapted to include all codons [26, 27]. The codon frequencies for yeast were based on 5,983 coding sequences and 2,929,341 codons, whereas those for *Xenopus laevis* were based on 72,864 coding sequences and 52,820,146 codons.

## Results

### Construction of pXOOY

**Fig 1:**
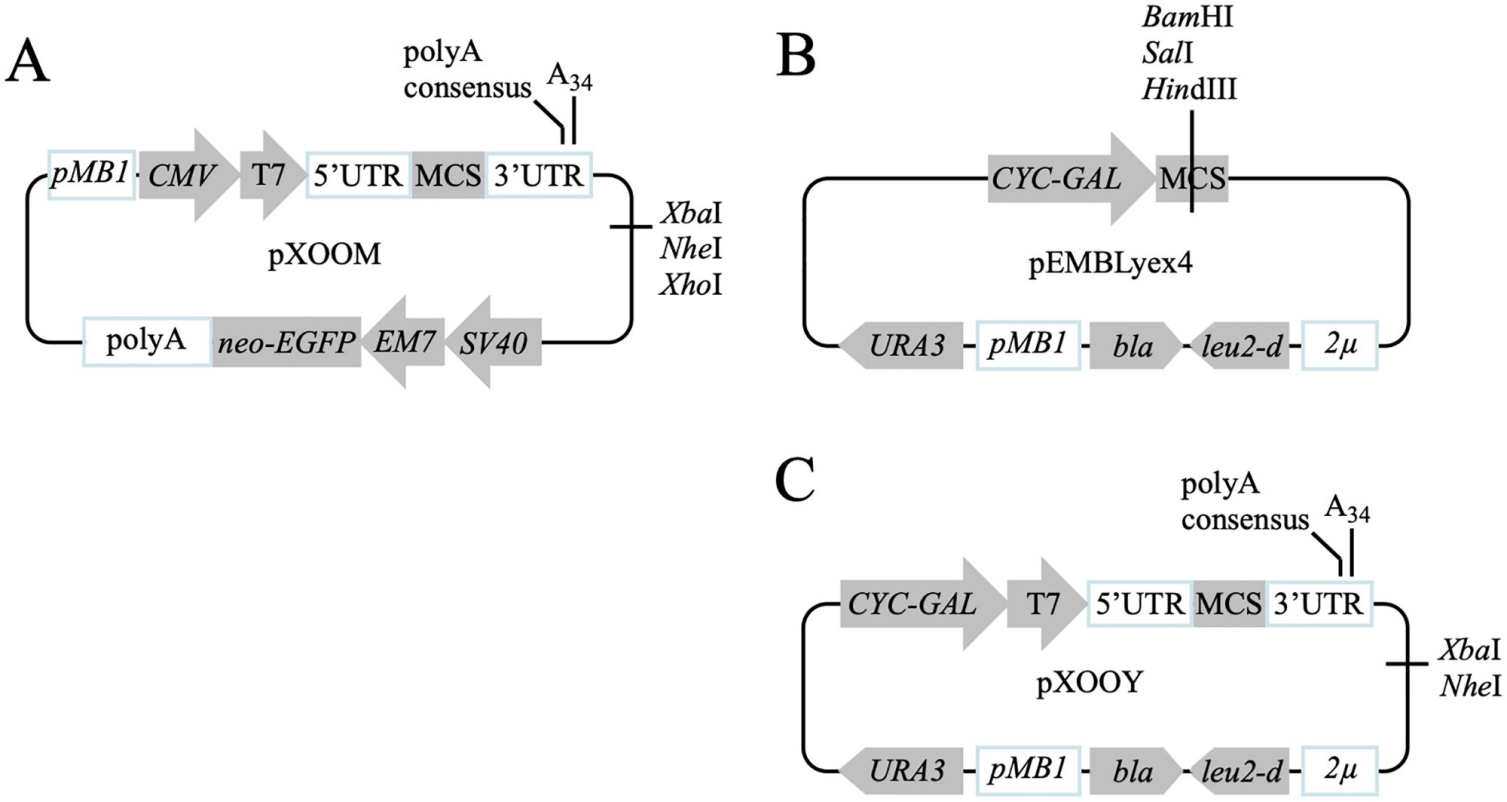
Plasmids used for creation of pXOOY. (A) The dual-function pXOOM plasmid created for heterologous protein expression in both mammalian cells and *Xenopus laevis* oocytes. *CMV*, cytomegalovirus early enhancer and promoter; T7, T7 RNA polymerase promoter; UTRs, both 5’ and 3’ regions from the *Xenopus laevis* β-globin gene; polyA consensus, native polyadenylation consensus sequence within the 3’UTR of *Xenopus laevis* β-globin gene; A_34_, synthetic polydT tail for *in vitro* transcription into a polyA tail; *SV40*, simian virus 40 early enhancer and promoter enabling expression of neo/kana-EGFP fusion gene; *EM7*, synthetic bacterial promoter directing expression of neo/kana-EGFP fusion gene; *neo/kana-EGFP*, neomycin resistance-enhanced GFP fusion gene; polyA, simian virus 40 fragment containing a polyadenylation signal. The *Xba*I, *Nhe*I and *Xho*I restriction sites can be used to linearize the plasmid prior to *in vitro* transcription. (B) pEMBLyex4 provides the backbone for the new pXOOY vector. *URA3*, encodes orotidine-5-phosphate decarboxylase; *CYC-GAL*, a fusion between the *CYC1* promoter and the *GAL10* upstream activating sequence containing two Gal4 binding sites; MCS, multicloning site; *2μ*, yeast two micron origin of replication; *leu2-d*, poorly expressed allele of the β-isopropylmalate dehydrogenase gene; *bla*, β-lactamase gene; *pMB1*, *E*. *coli* origin of replication. The restriction sites for *Bam*HI, *Hin*dIII and *Sal*I are used to linearize the plasmid at the MCS for insertion of PCR fragments into the vector using homologous recombination. (C) Plasmid map of the new dual-function pXOOY vector for expression of proteins in yeast and in *Xenopus laevis* oocytes. The expression cassette contains elements from pEMBLyex4 required for protein expression in yeast such as the *CYC-GAL* promoter and a yeast 5’UTR as well as the selective markers *URA3* and *leu2-d* and the *2μ* origin of replication. To enable *in vitro* transcription, it contains the pXOOM T7 promoter and the 3’UTR from the *Xenopus laevis* β-globin gene, which encodes a synthetic polyA tail comprised of 34 A-nucleotides.

We combined elements from the dual *Xenopus*-mammalian vector pXOOM shown in Fig 1A [4] and the yeast vector pEMBLyex4 [6] shown in Fig 1B for the construction of pXOOY (Fig 1C). The parental vectors have previously resulted in efficient expression of membrane proteins in oocytes [4] and yeast cells [15], respectively. Since the objective of protein production in yeast from pXOOY is to purify large amounts of protein for structural studies, it is important to ensure optimal expression in yeast. Therefore, pXOOY was designed to prioritize the features of pEMBLyex4 that give rise to high expression levels in yeast. To enable *in vitro* transcription from pXOOY, it was necessary to introduce a T7 promoter as well as a *Xenopus* 3’UTR containing a synthetic polyA tail of 34 adenosines to increase RNA stability and translatability in the oocytes.

**Fig 2:**
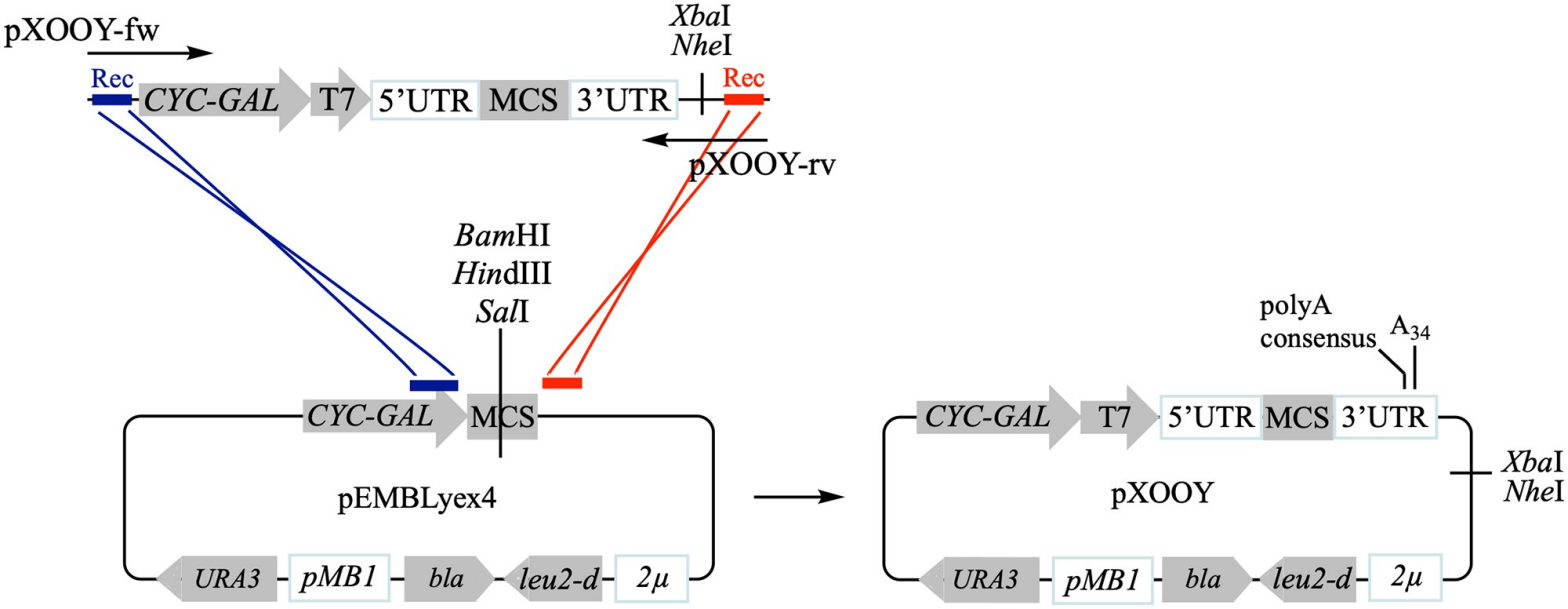
Illustration of how pXOOY was constructed by inserting a *Xenopus*-yeast dual expression cassette into pEMBLyex4. Both forward and reverse primers (pXOOY-fw and pXOOY-rv, respectively) used to amplify the *Xenopus*-yeast expression cassette are indicated by black arrows. Each primer contained a 5’ extension (**Rec** and **Rec**, respectively), which was identical to either side of the MCS in pEMBLyex4. These regions of homology direct correct insertion of the PCR amplified expression cassette. The PCR fragment and pEMBLyex4 linearized at its MCS with *Bam*HI, *Hin*dIII and *Sal*I to prevent plasmid recircularization were co-transformed into PAP1500 cells to assemble the final pXOOY plasmid by homologous recombination (shown to the right).

pXOOY was therefore constructed by inserting a dual *Xenopus*-yeast expression cassette into the backbone of pEMBLyex4 by homologous recombination as illustrated in Fig 2 [15].

**Fig 3:**
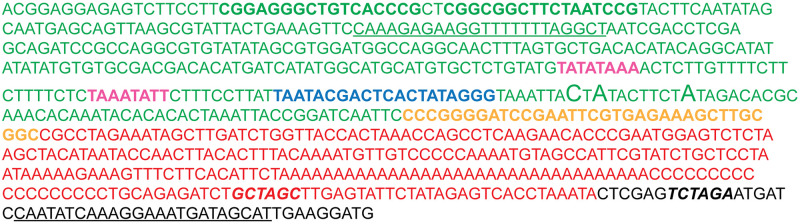
Detailed nucleotide sequence of the dual *Xenopus*-yeast expression cassette of pXOOY. Green nucleotides are the *CYC-GAL* promoter, nucleotides in bold green constitute the two Gal4 binding sites, nucleotides in bold pink are TATA boxes, bold blue nucleotides are the T7 promoter, yellow bold nucleotides are the MCS, nucleotides in red are 3’UTR from *Xenopus* β-globin, nucleotides written in bold italics in black and red are an *Xba*I site and an *Nhe*I site, respectively. Underlined nucleotides represent the recombination sites used to insert the pXOOY expression cassette into the pEMBLyex4 backbone. The larger letters indicate transcription start sites from the *CYC-GAL* promoter [29].

As previously mentioned, the design of the dual expression cassette should preserve the ability to direct the same high amount of recombinant protein as pEMBLyex4. We therefore maintained the distance between the TATA boxes of the promoter and the transcription start sites (TSS) as well as the distance between the TSS and the translational start codon. Therefore, 20 nucleotides between the last TATA box of the *CYC-GAL* promoter and the TSS were replaced with the 20-nucleotides long T7 promoter. We furthermore maintained the yeast 5’UTR rather than the 5’UTR from *Xenopus* β-globin found in pXOOM to preserve the yeast Kozak sequence. The yeast 3’UTR was replaced with the *Xenopus* β-globin 3’UTR to secure incorporation of a polyA tail by *in vitro* transcription for injection into the oocytes. Another feature of pEMBLyex4 responsible for high protein production in yeast is the *leu2-d* gene, which ensures a high plasmid copy number in yeast [28]. This feature was also transferred to pXOOY by using pEMBLyex4 as backbone. A detailed view of pXOOY and the DNA elements surrounding its multi-cloning site is provided in Fig 3.

**Fig 4:**
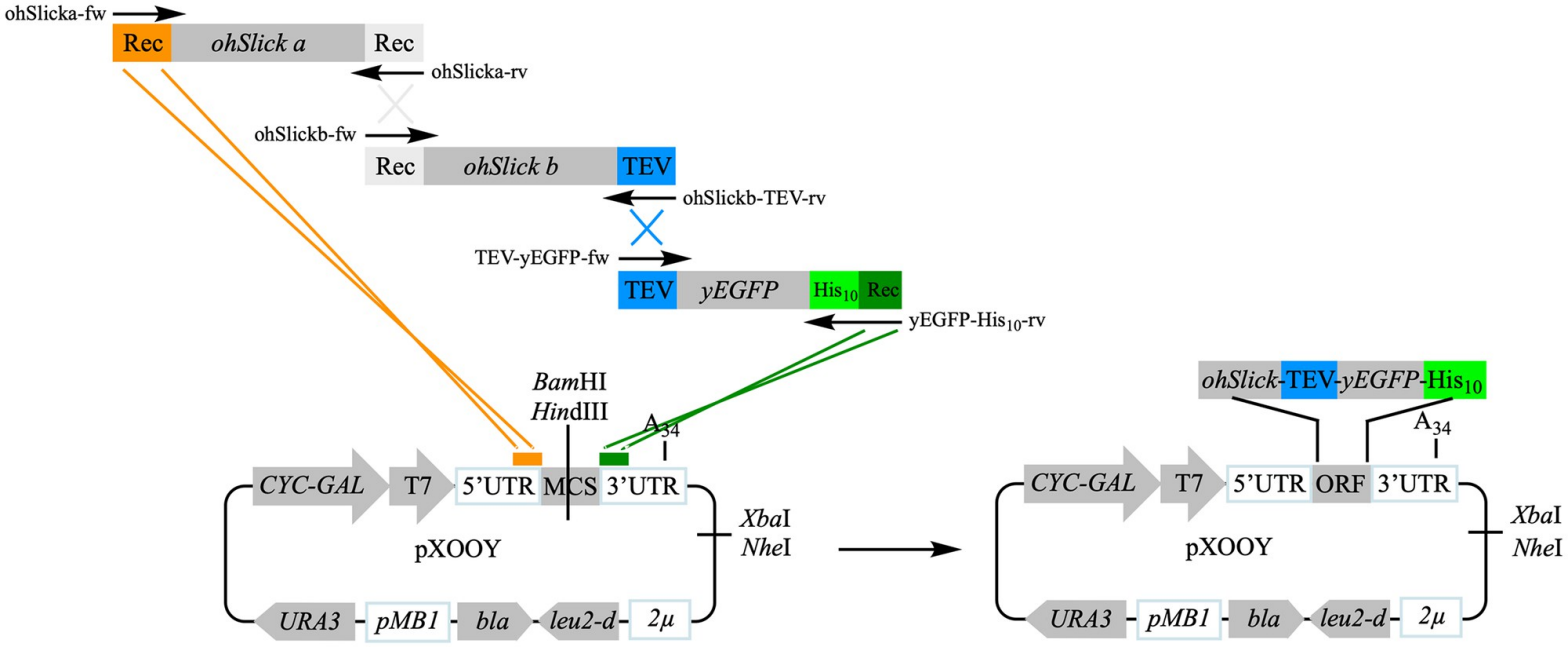
Illustration of how the ohSlick-TEV-yEGFP-His_10_ was assembled in pXOOY. The ohSlick cDNA was PCR amplified in two overlapping pieces (a and b) using the indicated primer pairs. A TEV site was introduced into the ohSlick b and yEGFP PCR fragments through 5’ extensions on the indicated PCR primers. A His_10_ tag was introduced to the yEGFP PCR fragment using a 5’ extension on the reverse PCR primer. The orange **Rec** and the green **Rec** are regions identical to either side of the MCS used for homologous recombination into pXOOY. All three PCR fragments were co-transformed into PAP1500 cells together with pXOOY linearized at its MCS with *Bam*HI and *Hin*dIII.

To construct the two final expression constructs, the backbone of pXOOY was linearized at the MCS with *Bam*HI and *Hin*dIII and transformed into PAP1500 yeast cells together with PCR fragments covering the entire open reading frame of ohERG or ohSlick sequences and yEGFP. Directed by the identity between the PCR fragments as shown in Fig 4 below for the ohSlick construct, the yeast cells assembled the PCR fragments into the pXOOY backbone by homologous recombination.

### Proof of concept: Expression in yeast

**Fig 5:**
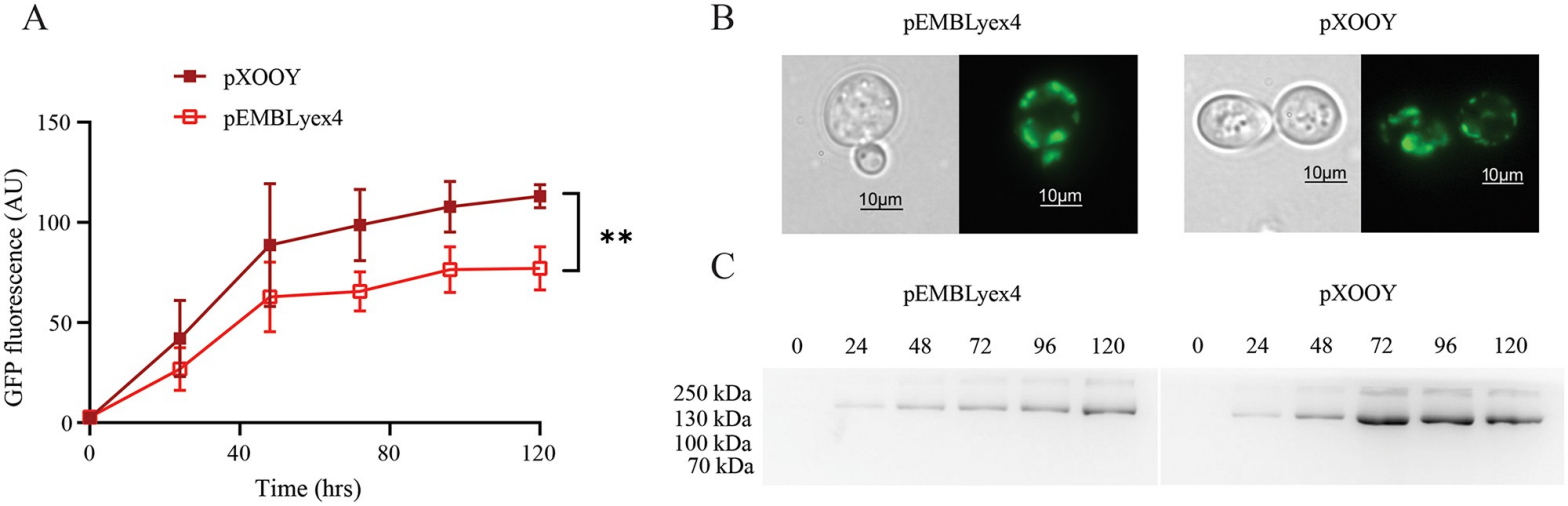
Accumulation of ohERG-TEV-yEGFP-His_10_ in *S*. *cerevisiae*. (A) Fluorescence in 1 OD_450_ unit as a function of time after induction of ohERG-TEV-yEGFP-His_10_ production with galactose. The dark red curve and the bright red curve show average fluorescence accumulation in PAP1500 cells expressing ohERG-TEV-yEGFP-His_10_ from pXOOY or pEMBLyex4, respectively. Averages were calculated from four repetitions of the experiment and error bars indicate ± S.D. The accumulation curves were compared using a two-way ANOVA test where **p = 0.0056. (B) Light microscopy (DIC) and fluorescence microscopy bio-imaging shown pairwise of yeast cells expressing either ohERG-TEV-yEGFP-His_10_ from pEMBLyex4 (left) or pXOOY (right). (C) In-gel fluorescence from crude yeast membranes isolated from cells harvested at the indicated time points after induction with galactose. The left gel shows 25 μg crude membrane samples from cells expressing ohERG-TEV-yEGFP-His_10_ from pEMBLyex4 and the right gel shows 25 μg membrane samples from cells expressing the same protein but from pXOOY. The two gels were imaged simultaneously. Uncropped gels can be found in S1, S2 Figs.

**Fig 6:**
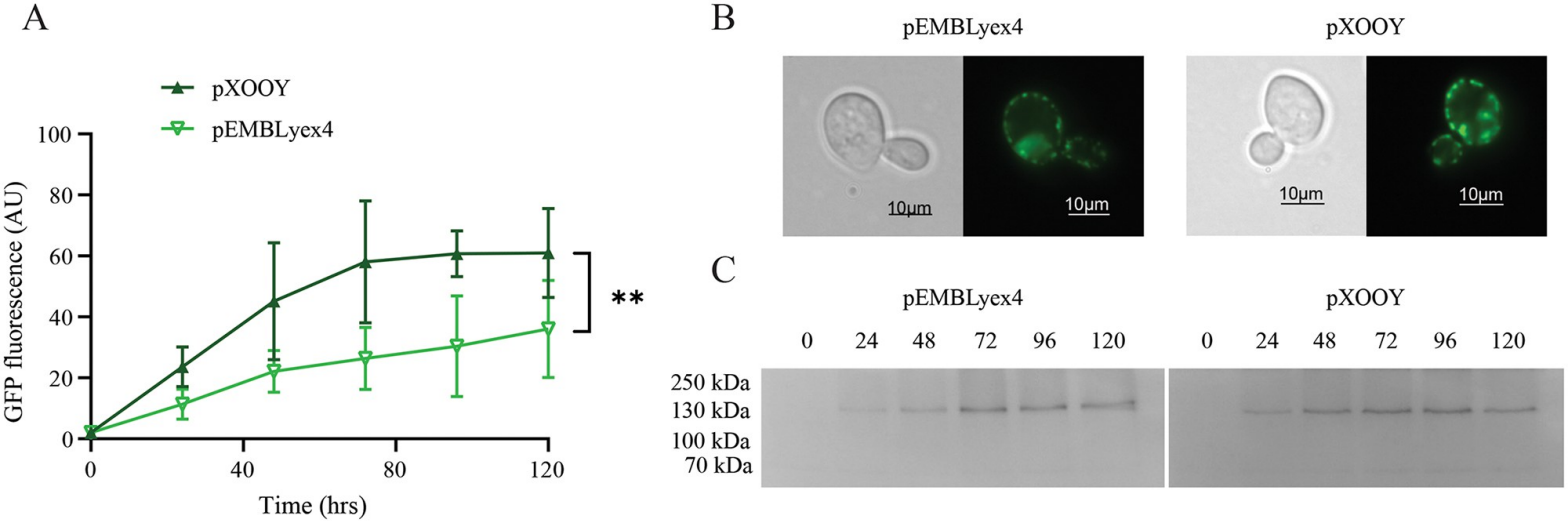
Accumulation of ohSlick-TEV-yEGFP-His_10_ in *S*. *cerevisiae*. (A) Fluorescence accumulation in 1 OD_450_ unit as a function of time after induction of ohSlick-TEV-yEGFP-His_10_ production. The bright green curve and the dark green curve show average fluorescence accumulation in PAP1500 cells expressing ohSlick-TEV-yEGFP-His_10_ from pEMBLyex4 or pXOOY, respectively. Averages were calculated from four repetitions of the experiment and error bars indicate ± S.D. The accumulation curves were compared using a two-way ANOVA test where **p = 0.0012. (B) Light microscopy and fluorescence microscopy bio-imaging of yeast cells expressing either ohSlick-TEV-yEGFP-His_10_ from pEMBLyex4 (left) or pXOOY (right). (C) In-gel fluorescence of an SDS-PAGE gel of 25 μg yeast membranes purified from yeast cells harvested at the indicated time points after induction with galactose. The left gel shows purified membrane samples from cells expressing ohSlick-TEV-yEGFP-His_10_ from pEMBLyex4 and the right gel shows membrane samples from cells expressing the same fusion protein but from pXOOY. The two gels were imaged with the same exposure time. Uncropped gels can be found in S3, S4 Figs.

To demonstrate that expression from pXOOY was able to generate protein amounts comparable with those from pEMBLyex4, we expressed two ion channels from both pXOOY and pEMBLyex4 in PAP1500 yeast cells. The results are summarized in Fig 5 for ohERG-TEV-yEGFP-His_10_ and in Fig 6 for ohSlick-TEV-yEGFP-His_10_, respectively. Time-dependent accumulation of the two fusion proteins was investigated four times for each construct in each vector by quantifying the GFP fluorescence emitted from 1 OD_450_ unit of cells. Averaging the four experiments shows that expression of both ohERG-TEV-yEGFP-His_10_ and ohSlick-TEV-yEGFP-His_10_ from pXOOY exceeds accumulation levels from pEMBLyex4 (Figs 5A and 6A). This suggests that insertion of T7 promoter and exchanging the 3’UTR somehow enhances expression in yeast cells. To verify that the measured GFP fluorescence in Figs 5A and 6A originated from accumulation of membrane integrated GFP fusions, we compared accumulation of the ohERG and ohSlick constructs when expressed from pXOOY or pEMBLyex4. Live cell bioimaging in Fig 5B revealed that ohERG-TEV-yEGFP-His_10_ expressed from pEMBLyex4 localized mainly to intracellular compartments, which was also observed when the construct was expressed from pXOOY. Fig 6B shows that ohSlick-TEV-yEGFP-His_10_ accumulated in both plasma and intracellular membranes when expressed from either plasmid.

At each time point, 25 μg of purified yeast membrane samples from cultures expressing each construct were analyzed by in-gel fluorescence (Figs 5C and 6C). Since only one fluorescent band of expected size was present in each lane, yEGFP must be correctly folded and since it is attached C-terminally to ohERG or ohSlick these must likewise have obtained their correct three dimensional structure [30]. Furthermore, the absence of smaller fluorescent bands suggests that only full-length protein has accumulated in the membranes.

### Proof of concept: Expression in *Xenopus laevis* oocytes

**Fig 7:**
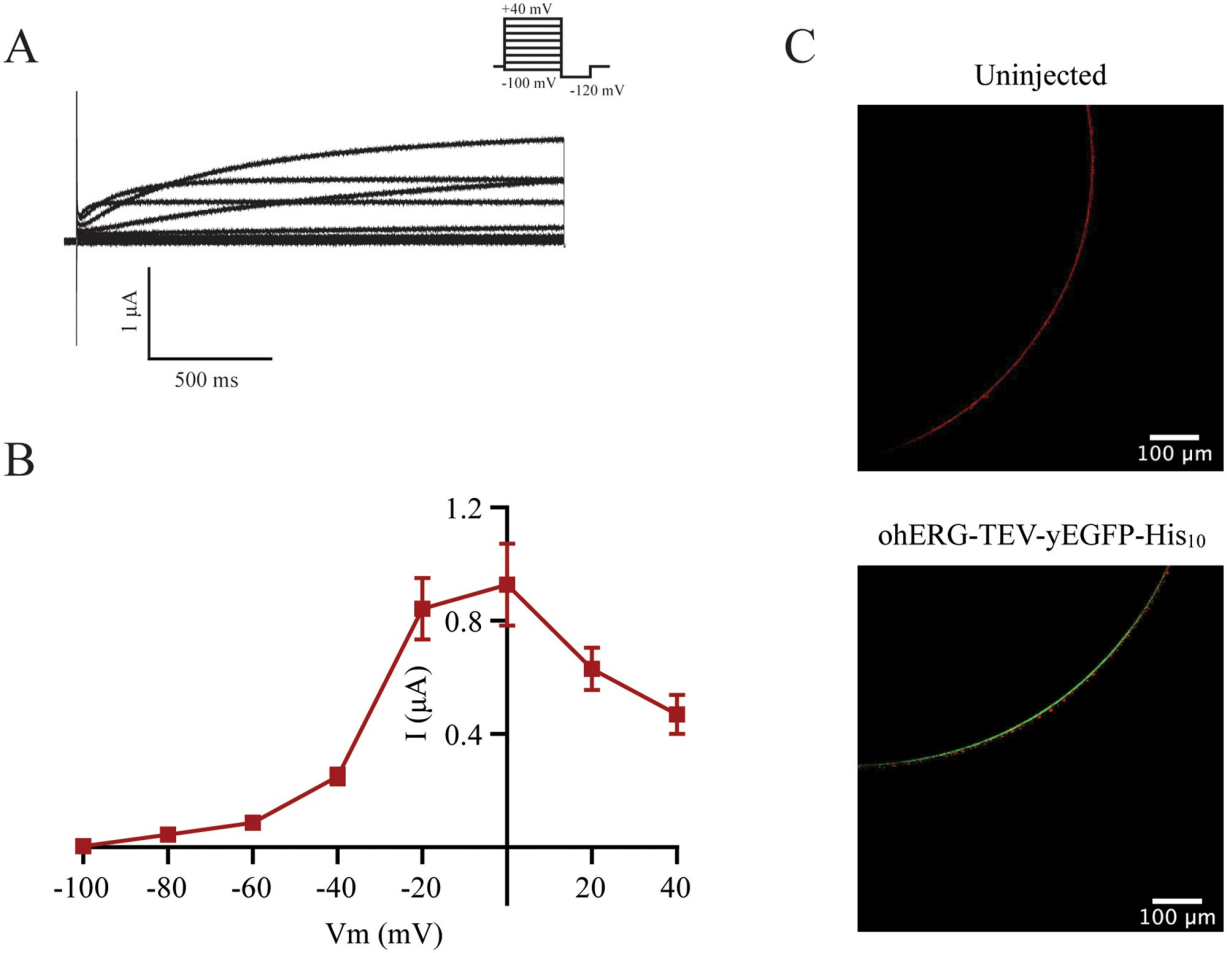
Expression of ohERG-TEV-yEGFP-His_10_ in *Xenopus laevis* oocytes. (A) Representative current traces of ohERG-TEV-yEGFP-His_10_ measured in *Xenopus laevis* oocytes as described in the materials and methods section 5 days after injection of 25 ng of polyA RNA *in vitro* transcribed from pXOOY. The tail currents recorded at -120 mV are shown in S5 Fig. (B) I-V relation of ohERG-TEV-yEGFP-His_10_ given as the average ± S.E.M (n = 13). (C) Representative confocal images of oocytes incubated with FM 4–64. The upper image shows an uninjected oocyte, and the lower image shows an oocyte expressing ohERG-TEV-yEGFP-His_10_, which colocalized with FM4-64.

**Fig 8:**
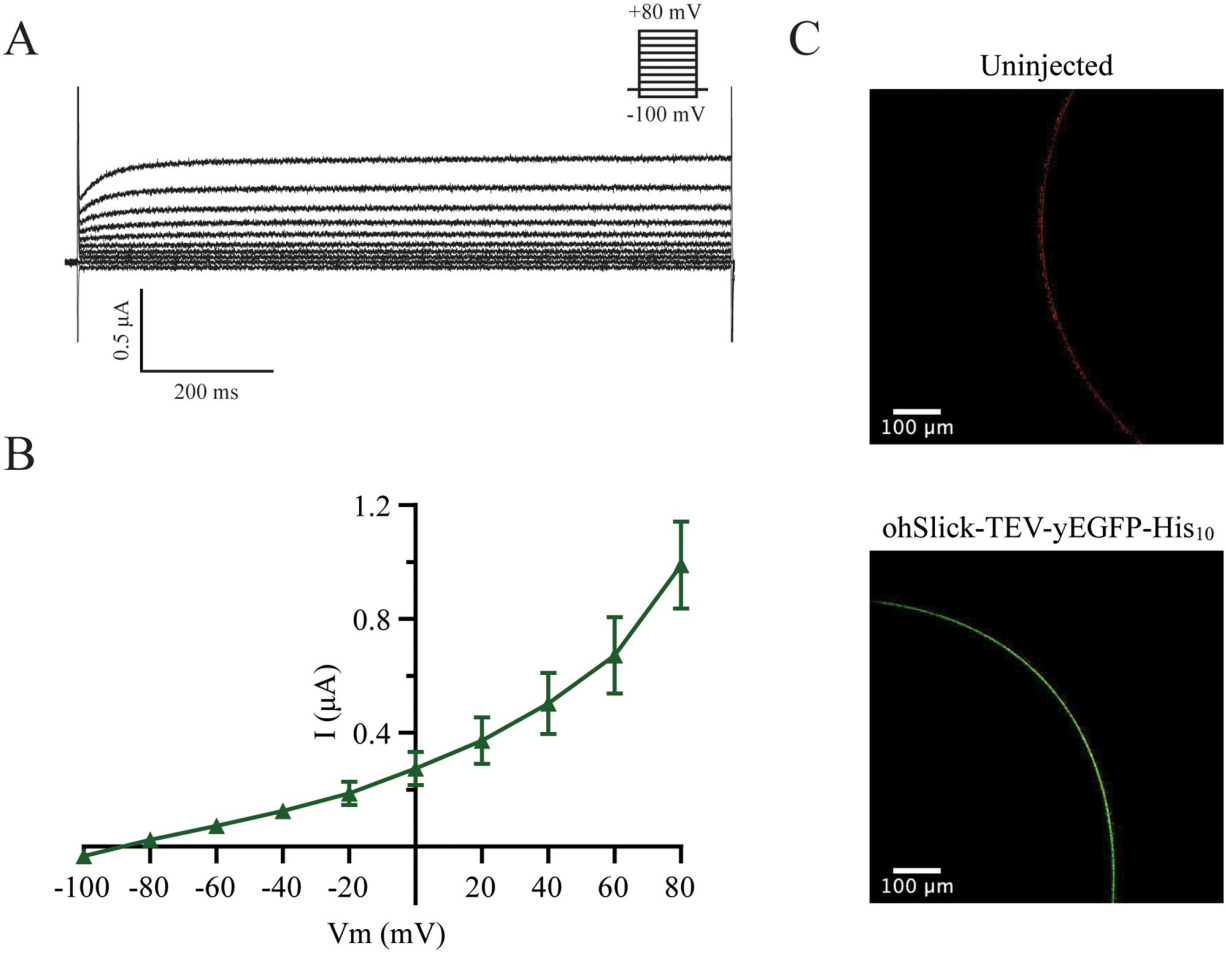
Expression of ohSlick-TEV-yEGFP-His_10_ in *Xenopus laevis* oocytes. (A) Representative traces of ohSlick-TEV-yEGFP-His_10_ expressed and measured in *Xenopus laevis* oocytes 5 days after injection of 25 ng of polyA RNA *in vitro* transcribed from pXOOY. (B) I-V relation of ohSlick-TEV-yEGFP-His_10_ shown as the average ± S.E.M (n = 11). (C) Representative confocal images of oocytes incubated with FM 4–64. The upper image shows an uninjected control oocyte, and the lower image shows an oocyte expressing ohSlick-TEV-yEGFP-His_10_, which colocalizes with the FM4-64 dye.

To demonstrate that ohERG-TEV-yEGFP-His_10_ and ohSlick-TEV-yEGFP-His_10_ produced from pXOOY give rise to hERG and hSlick currents with full preservation of electrophysiological characteristics, the constructs were expressed in *Xenopus laevis* oocytes. Figs 7A and 8A show representative currents measured for ohERG-TEV-yEGFP-His_10_ and ohSlick-TEV-yEGFP-His_10_, respectively, and Figs 7B and 8B show their current-voltage relationship. Fig 7A and 7B show currents and current-voltage relationships that are typical of heterologously expressed hERG channels [31]. When we have previously expressed hERG from pXOOM, the maximal current measured at 0 mV are typically around 2–3 μA (see e.g. ref [4]). In comparison, expression of ohERG-TEV-yEGFP-His_10_ gives maximal currents of approximately 0.9 μA at 0 mV. The measured currents follow the well-known activation and inactivation kinetics for hERG channels, suggesting that fully functional channels are expressed. Characteristic hERG tail currents are shown in S5 Fig. This indicates that attaching a large TEV-yEGFP-His_10_ moiety to the C-terminus of hERG does not affect the voltage dependence of the channel. Unlike hERG, Slick does not inactivate at depolarized membrane potentials and that is also observed for ohSlick-TEV-yEGFP-His_10_ and summarized in Fig 8A and 8B. In our experience, when hSlick is expressed from pXOOM, it typically gives currents ranging from 1–3 μA at +80 mV (see e.g. ref [11, 23]). When expressed from pXOOY, ohSlick gives currents around 1 μA at +80 mV (Fig 8B).

As for the yeast cells, localization of GFP-tagged ohERG and ohSlick was studied using live cell bioimaging of oocytes expressing ohERG-TEV-yEGFP-His_10_ or ohSlick-TEV-yEGFP-His_10_ from pXOOY. The confocal images in Figs 7C and 8C show that both constructs and the membrane dye FM4-64 clearly co-localize at the plasma membrane. Furthermore, they show a clear difference between non-expressing oocytes and oocytes expressing either of these constructs in terms of GFP fluorescence. Since the ion channels are C-terminally GFP-tagged, it can be inferred that if GFP localizes to the plasma membrane so does the channel itself.

## Discussion

The aim of this paper is to demonstrate how a dual *Xenopus*-yeast expression vector for production of membrane proteins can be constructed. The goal is that the vector can be used to produce membrane protein recombinantly in yeast in yields high enough for X-ray crystallography or cryo-EM, and that polyA RNA can be generated by *in vitro* transcription for subsequent injection into *Xenopus laevis* oocytes to verify protein functionality and to characterize amino acid substituted variants.

Although the elements required for efficient production of a recombinant protein are the same in all eukaryotic cells, their specific nucleotide sequences are host specific. The elements that define an expression cassette include a promoter region, a 5’UTR of the mRNA, a Kozak sequence for optimal translation initiation, a codon optimized cDNA, a 3’UTR of the mRNA, and a transcriptional terminator including a polyadenylation sequence. When designing an expression cassette for recombinant protein production, it is important to adapt the structure and nucleotide sequences of these elements to fit the host organism. An inherent problem in designing a dual-function vector is that it is necessary to compromise on the origin of these elements as experience suggests they cannot be optimal in two organisms as phylogenetically distinct as *S*. *cerevisiae* and *Xenopus laevis*.

We aimed at generating a dual-function expression plasmid that delivers the same amount of prime quality membrane protein as the original pEMBLyex4 vector [2, 32, 33]. Therefore, our strategy was to supply the yeast vector with functions required for *in vitro* transcription and generation of polyA RNA. To do so, we decided to substitute a 20 nucleotides long sequence upstream from the transcriptional start site of the yeast *CYC-GAL* promoter in pEMBLyex4 with the 20 nucleotides long T7 promoter sequence. This was done to preserve the transcriptional start site in yeast and thus maintain the nucleotide sequence of the 5’UTR in yeast. The Kozak sequence used in pEMBLyex4 and in pXOOY has the nucleotide sequence UUUAAAACGAAUGG for expression of ohSlick, and UAAGAUAAUUAUGC for expression of ohERG, while the consensus sequence in *Xenopus laevis* is MM**A**MMAUG**R** (where M = A or C, R = A or G) [34]. The two Kozak sequences used for expression of ohSlick and ohERG do therefore not conform to the consensus sequence in *Xenopus*, which may explain why the maximum current amplitudes were apparently lower as compared to those observed earlier from the *Xenopus* oocyte optimized vector pXOOM (4). However, we did not perform a side-by-side comparison of pXOOM and pXOOY so the difference in current amplitude might alternatively reflect the quality of the oocytes and the RNA generated by *in vitro* transcription. Nevertheless, the expression levels were sufficient to record channel activity and perform full electrophysiological characterization.

Another important issue in recombinant protein production is optimizing the cDNA sequence to match the codon usage in the host. The lower currents observed in the oocytes could therefore be related to the yeast codon optimization of the ion channel sequences. Codon usage frequencies of *Xenopus laevis* and yeast presented in S6 Fig were accessed using the online database CoCoPUT (Codon and Codon-Pair Usage Table) according to the Materials and Methods section. When inspecting this graphical comparison, it becomes evident that some of the yeast codons are less common in *Xenopus*. This is particularly noticeable for codons TTA, and TTG, which encode leucine, and ATT, AAT, and GGT encoding isoleucine, asparagine, and glycine, respectively. These five codons appear almost twice as frequent in yeast as in *Xenopus*. Nevertheless, there are no very frequent codons in yeast that are extremely rare in *Xenopus*. Despite a number of suboptimal codons for oocyte expression, the two ion channel proteins were expressed to an acceptable level.

As poly-adenylation is required for stability of the *in vitro* generated polyA RNA and its translatability in the oocytes, we exchanged the yeast 3’UTR and transcriptional terminator for those from the *Xenopus* β-globin gene. This did not negatively impact expression of the two model proteins in yeast, in contrast, the accumulation of recombinant protein produced from pXOOY increased slightly compared to that obtained from pEMBLyex4. So at least for our model proteins, this *Xenopus* element seems to work as well as the yeast element.

We used GFP-tagged versions of ohERG and ohSlick potassium channels to simplify quantification of channel accumulation in yeast and determine their localization in both organisms. This may potentially interfere with assembly, targeting, and function of these two homo-tetrameric channels in *Xenopus* oocytes. However, as seen in Figs 7B and 8B this was not the case as the I-V curves obtained for the GFP tagged channels are similar to those previously published for the non-tagged channels [22, 23]. Efficient targeting was also confirmed by the fact that both channels localize to the plasma membrane stained with FM4-64 (Figs 7C and 8C).

Our results therefore demonstrate that it is possible to design a *Xenopus*-yeast dual expression vector without compromising recombinant membrane protein production in *S*. *cerevisiae* while preserving the ability to observe characteristic channel activity in *Xenopus laevis* oocytes. Use of the dual-function vector will save a large amount of time as only half as many plasmids must be constructed for structural studies in the future.

## Supporting information

S1 Fig Uncropped SDS-PAGE gel of ohERG-TEV-yEGFP-His_10_ expressed from pEMBLyex4 (left) and pXOOY (right) showing in-gel fluorescence. (PDF)

S2 Fig Uncropped gel of ohERG-TEV-yEGFP-His_10_ expressed from pEMBLyex4 (left) and pXOOY (right) showing the molecular weight marker. (PDF)

S3 Fig Uncropped gel of ohSlick-TEV-yEGFP-His_10_ expressed from pEMBLyex4 showing the in-gel fluorescence. The molecular marker is visible.
(PDF)

S4 Fig Uncropped gel of ohSlick-TEV-yEGFP-His_10_ expressed from pXOOY showing the in-gel fluorescence. The molecular marker is visible.
(PDF)

S5 Fig Representative pXOOY-expressed ohERG currents showing the characteristic ohERG tail current. (TIF)

S6 Fig CoCoPUT codon frequency comparison between *S*. *cerevisiae* and *Xenopus laevis*. (TIF)

S1 Table PCR primer sequences. (PDF)

S2 Table Sequencing primers. (PDF)

S3 Table Accumulation of ohERG and ohSlick expressed from pEMBLyex4 and pXOOY as GFP fluorescence per 1 OD_450_. (PDF)

S4 Table Baseline corrected TEVC data recorded for pXOOY-based ohERG and ohSlick currents. (PDF)

S1 File Annotated sequence of pXOOY. (DNA)

S2 File Annotated sequence of ohERG-TEV-yEGFP-His_10_ in pXOOY. (DNA)

S3 File Annotated sequence of ohSlick-TEV-yEGFP-His_10_ in pXOOY. (DNA)
