# Supplementary material for: pXOOY: A dual-function vector for expression of membrane proteins in Saccharomyces cerevisiae and Xenopus laevis oocytes
Source: PLoS One. 2023 Feb 21;18(2):e0281868. doi: 10.1371/journal.pone.0281868 (PMC9942955; doi:10.1371/journal.pone.0281868)
Supplement: S1 Fig — (PDF) [file pone.0281868.s001.pdf]

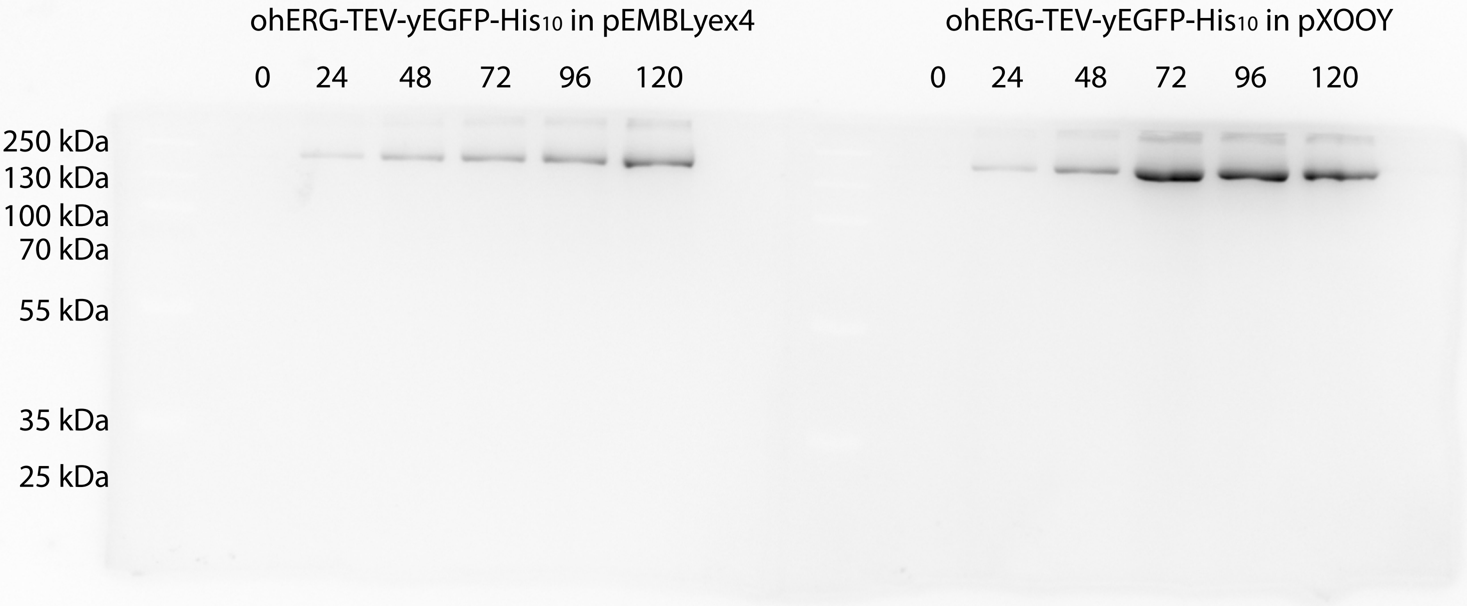
ohERG-TEV-yEGFP-His10 in pEMBLyex4
0 24 48 72 96 120
ohERG-TEV-yEGFP-His10 in pXOOY
0 24 48 72 96 120
250 kDa
130 kDa
100 kDa
70 kDa
55 kDa
35 kDa
25 kDa
