# Supplementary figures and images for: pXOOY: A dual-function vector for expression of membrane proteins in Saccharomyces cerevisiae and Xenopus laevis oocytes
Source: PLoS One. 2023 Feb 21;18(2):e0281868. doi: 10.1371/journal.pone.0281868 (PMC9942955; doi:10.1371/journal.pone.0281868)

ohSlick-TEV-yEGFP-His<sub>10</sub> in pEMBLyex4

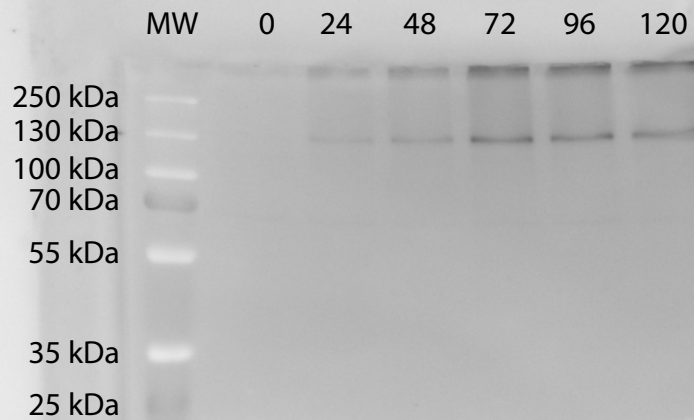

Supplement: S3 Fig — The molecular marker is visible. (PDF) [file pone.0281868.s003.pdf]

ohSlick-TEV-yEGFP-His<sub>10</sub> in pXOOY

0 24 48 72 96 120

250 kDa

130 kDa

100 kDa

70 kDa

55 kDa

35 kDa

25 kDa

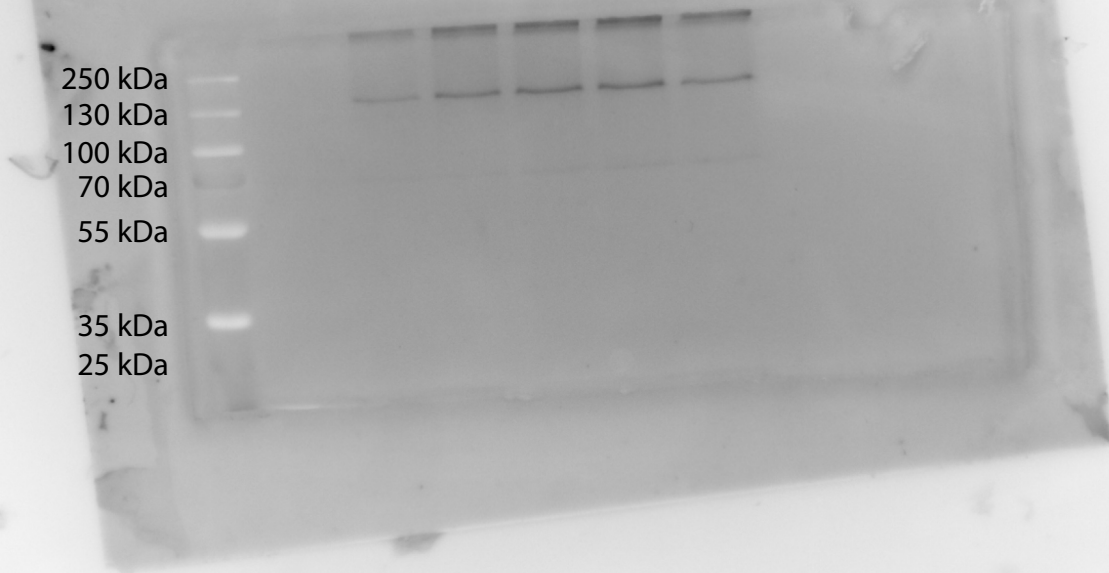

Supplement: S4 Fig — The molecular marker is visible. (PDF) [file pone.0281868.s004.pdf]

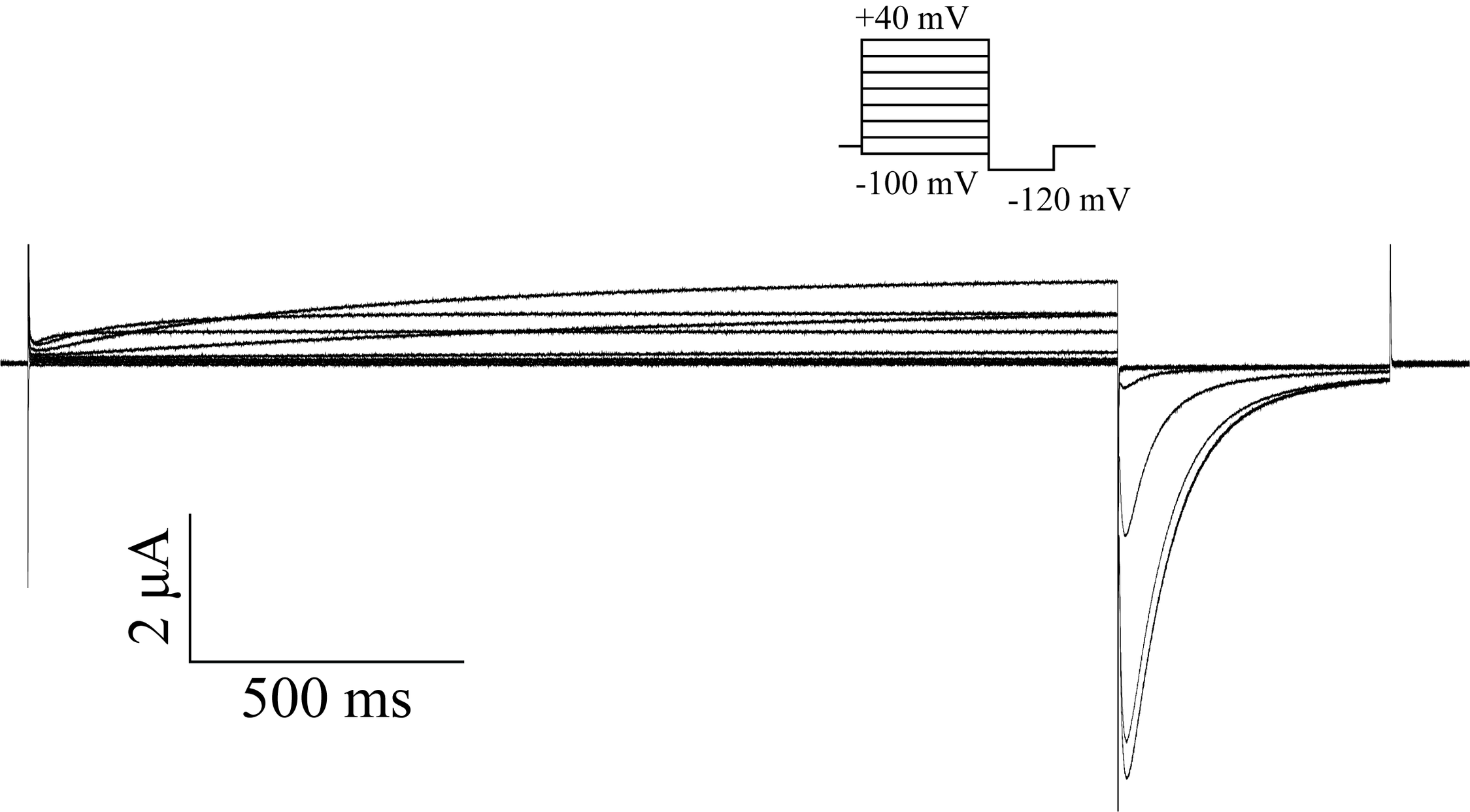

Supplement: S5 Fig — (TIF) [file pone.0281868.s005.tif]

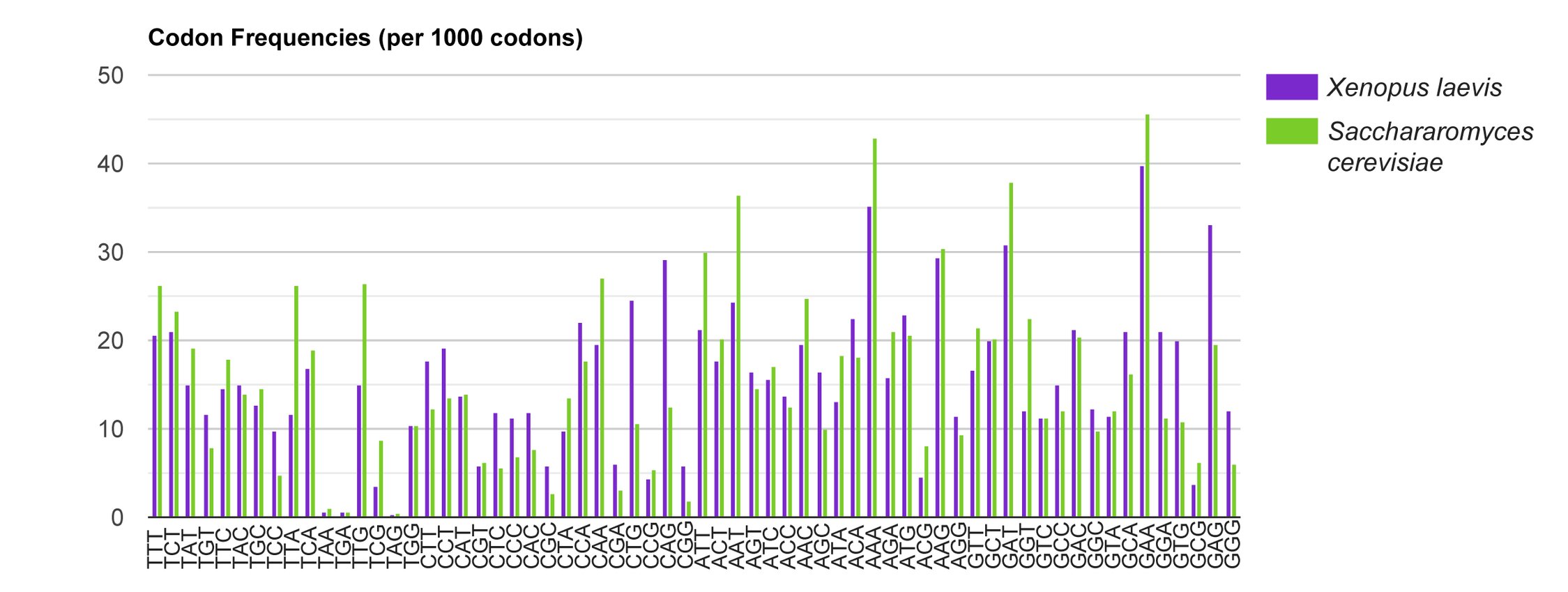

Supplement: S6 Fig — (TIF) [file pone.0281868.s006.tif]
