# Supplementary material for: pXOOY: A dual-function vector for expression of membrane proteins in Saccharomyces cerevisiae and Xenopus laevis oocytes
Source: PLoS One. 2023 Feb 21;18(2):e0281868. doi: 10.1371/journal.pone.0281868 (PMC9942955; doi:10.1371/journal.pone.0281868)
Supplement: S1 Table — (PDF) [file pone.0281868.s007.pdf]

S1 Table: PCR primer sequences

pXOOY expression cassette PCR primers

pXOOY-fw	5’-TAGCAATGAGCAGTTAAGCGTAT TACTGAAAGTTCCAAAGAGAAGGT TTTTTTAGGCT-3’
pXOOY-rv	5’-CTGCCACTCCTCAATTGGATTAGT CTCATCCTTCAATGCTATCATTTCCT TTGATATTG-3’

ohERG PCR primers

ohERG-fw	5 ′-ACACAAATACACACACTAAATTA CCGGATCAATTCTTTAAAACGAATG CCAGTTAGAAGAGGTC-3 ′
TEV-ohERG-rv	5 ′-AAAT TGACTTTGAAAATACAAA TTTTCACTACCTGGGTCACTACCG-3 ′

ohSlick PCR primers

ohSlicka-fw	5’-ACACAAATACACACACTAAATTA CCGGATCAATTCTAAGATAATTATG GTAGATTTAGAAAGTGAAGT-3’
ohSlicka-rv	5’-ATAAAAACTTCTGGAAACGTT TGA- 3’
TEV-ohSlickb-fw	5’-GAAAGTCAAACGTTTCCAGAAG TTTTTATCACGGTCCTAGTAGATTA CC-3’
TEV-ohSlickb-rv	5’-AAATTGACTTTGAAAATACAAA TTTTCTAATTGTGTTTCTTCTCTTG AATC-3’

yEGFP-His_{10} PCR primers

yEGFP-His10-fw	5'-GAAAATTTGTATTTTCA AAGTCAATTT ATGTCTAAAGGTGA AGAATTATTCACT-3'
TEV-yEGFP-His10-rv	5'-CTTCAATGCTATCATTTCCTTTGAT ATTGGATCATTCAATGGTGATGGTG ATGGTGATGGTGATGGTGTTTGTACA ATTCATCCATACCA-3'
