# Supplementary material for: pXOOY: A dual-function vector for expression of membrane proteins in Saccharomyces cerevisiae and Xenopus laevis oocytes
Source: PLoS One. 2023 Feb 21;18(2):e0281868. doi: 10.1371/journal.pone.0281868 (PMC9942955; doi:10.1371/journal.pone.0281868)
Supplement: S2 Table — (PDF) [file pone.0281868.s008.pdf]

S2 Table: Sequencing primers

ohERG sequencing primers

Galsekv	5’ GCGAGGCACATCTGCGTTTC 3’
CYC-GAL	5’-CTATACTTCTATAGACACGC-3’
ohERG_451	5′-CCTACAAGTTGGTTAGCACC-3′
ohERG_815	5′-CTAGAGAATCATGCGCTTCC-3′
ohERG_1347	5′- CCAACCATTAGCTGTAGTCG – 3′
ohERG_1825	5′- GACAAGTACGTTACCGCTTT – 3′
ohERG_2317	5′- GGTGACTTATTGACCGCTTT – 3′
ohERG_2754	5′- CAGTAGAGGTAGACCAGGTG – 3′
GFPsekv2	5’-GTAGCATCACCTTCACCTTC-3’

ohSlick sequencing primers

Galsekv	5’ GCGAGGCACATCTGCGTTTC 3’
CYC-GAL	5’-CTATACTTCTATAGACACGC-3’
ohSlick_526	5’-CCTTGGAAAACATGATCAAC-3’
ohSlick_1125	5’-GATGACGCTGAAGCATGTTT-3’
ohSlick_1651	5’-TCTACATCAACATCACAAAGG-3’
ohSlick_2112	5’-CATAACTACTACGAAGATGCT-3’
ohSlick_2638	5’-TGCCATTTGCAGCCGGTA-3’
ohSlick_3237	5’-TTGAGTTACATCTTGATCAAC-3’
GFPsekv2	5’-GTAGCATCACCTTCACCTTC-3’
