# Supplementary material for: pXOOY: A dual-function vector for expression of membrane proteins in Saccharomyces cerevisiae and Xenopus laevis oocytes
Source: PLoS One. 2023 Feb 21;18(2):e0281868. doi: 10.1371/journal.pone.0281868 (PMC9942955; doi:10.1371/journal.pone.0281868)
Supplement: S3 Table — (PDF) [file pone.0281868.s009.pdf]

S3 Table: Accumulation of ohERG and ohSlick expressed from pEMBLyex4 and pXOOY as GFP fluorescence per 1 OD_{450}

**Time**	**ohERG-TEV-yEGFP-His$_{10}$ from pEMBLyex4**			
**0**	2.087	2.08	3.086	3.792
**24**	11.303	29.094	35.364	31.724
**48**	45.546	53.871	85.741	66.271
**72**	61.503	62.032	80.202	58.732
**96**	76.041	67.737	92.647	69.337
**120**	77.98	69.565	91.915	68.795

**Time**	**ohERG-TEV-yEGFP-His$_{10}$ from pXOOY**			
**0**	1.042	3.054	3.213	2.604
**24**	14.013	53.104	54.854	46.404
**48**	43.946	106.261	110.261	94.271
**72**	72.703	111.772	102.772	107.672
**96**	89.561	115.007	117.807	108.707
**120**	107.38	114.055	120.755	110.155

**Time**	**ohSlick-TEV-yEGFP-His$_{10}$ from pEMBLyex4**			
**0**	1.593	2.916	1.172	2.353
**24**	9.148	8.958	18.713	8.688
**48**	19.557	19.017	32.393	17.627
**72**	22.886	21.246	41.571	19.936
**96**	24.529	21.669	55.052	20.309
**120**	31.08	24.97	59.773	28.51

**Time**	**ohSlick-TEV-yEGFP-His$_{10}$ from pXOOY**			
**0**	2.234	0.106	2.908	2.906
**24**	30.084	14.596	23.694	26.024
**48**	57.951	16.655	54.391	51.491
**72**	70.062	28.247	69.472	64.482
**96**	67.897	51.514	57.807	65.777
**120**	70.355	39.243	67.855	66.335
