# Supplementary material for: pXOOY: A dual-function vector for expression of membrane proteins in Saccharomyces cerevisiae and Xenopus laevis oocytes
Source: PLoS One. 2023 Feb 21;18(2):e0281868. doi: 10.1371/journal.pone.0281868 (PMC9942955; doi:10.1371/journal.pone.0281868)
Supplement: S4 Table — (PDF) [file pone.0281868.s010.pdf]

S4 Table: Baseline corrected TEVC data of ohERG and ohSlick from pXOOY

Vm (mV)	ohERG-TEV-yEGFP-His$_{10}$												
-100	-0.014254	0.0044504	-0.028247	-0.01835	0.012094	0.0156883	0.0154746	0.0061296	0.00782646	0.0106388	0.007989	0.022719	0.008442
-80	0.0546051	0.0378843	0.0666352	0.060809	0.027717	0.0319191	0.0296339	0.0399945	0.03755585	0.0472457	0.06116	0.046354	0.032506
-60	0.127797	0.073671	0.168391	0.143082	0.062487	0.0559715	0.0441015	0.0580269	0.0638842	0.0884021	0.119584	0.076332	0.047252
-40	0.425023	0.342447	0.434572	0.306396	0.262928	0.217342	0.178081	0.175089	0.182982	0.17193	0.257399	0.151746	0.144483
-20	1.51697	1.67814	1.26762	0.734807	0.586992	0.572973	0.554146	0.511626	0.763142	0.681107	0.929791	0.621227	0.530318
0	1.5951	1.92335	1.29479	0.895454	0.470059	0.503574	0.561246	0.565627	0.100448	1.1321	1.42271	0.930799	0.659905
20	0.962447	1.03307	0.946887	0.763572	0.283851	0.301355	0.335868	0.360229	0.625374	0.694487	0.881325	0.574233	0.423294
40	0.739367	0.626761	0.940352	0.780391	0.183369	0.199359	0.215586	0.262303	0.38808	0.457015	0.645402	0.387561	0.278085

Vm (mV)	ohSlick-TEV-yEGFP-His$_{10}$										
-100	-0.0148122	-0.01078	0.012776	0.006193	-0.13138	-0.02149	-0.00784	-0.0133235	-0.00197	-0.02982	-0.12106
-80	0.0279496	0.014812	0.0295	0.015349	0.015673	0.022973	0.024145	0.0260516	0.028334	0.034565	0.024142
-60	0.0637892	0.044176	0.054573	0.032373	0.168676	0.052643	0.042156	0.0484932	0.052089	0.094737	0.142343
-40	0.114701	0.084072	0.072883	0.050504	0.326387	0.078371	0.061125	0.0972469	0.078777	0.15617	0.252506
-20	0.181394	0.127243	0.098105	0.069891	0.496118	0.116899	0.093587	0.138111	0.123536	0.2116	0.394903
0	0.304394	0.194531	0.12593	0.105316	0.688454	0.167465	0.138526	0.202384	0.187952	0.293367	0.603376
20	0.424045	0.269448	0.151212	0.142685	0.903509	0.230238	0.185738	0.267029	0.246444	0.400775	0.873402
40	0.602759	0.365281	0.17386	0.189927	1.1336	0.343153	0.256019	0.367104	0.338835	0.533593	1.22405
60	0.817387	0.49204	0.243602	0.330369	1.40468	0.492732	0.341737	0.486458	0.451002	0.712818	1.6137
80	1.16145	0.80451	0.506981	0.77	1.79153	0.965839	0.537977	0.672057	0.642545	0.960209	2.06901
